# Wnt/β-catenin, an oncogenic pathway targeted by *H. pylori* in gastric carcinogenesis

**DOI:** 10.18632/oncotarget.5758

**Published:** 2015-09-21

**Authors:** Xiaowen Song, Na Xin, Wei Wang, Chenghai Zhao

**Affiliations:** ^1^ Department of Pathophysiology, College of Basic Medical Science, China Medical University, Shenyang, China

**Keywords:** Wnt, β-catenin, Helicobacter Pylori, gastric cancer

## Abstract

A section of gastric cancers presents nuclear β-catenin accumulation correlated with *H. pylori* infection. *H. pylori* stimulate Wnt/β-catenin pathway by activating oncogenic c-Met and epidermal growth factor receptor (EGFR), or by inhibiting tumor suppressor Runx3 and Trefoil factor 1 (TFF1). *H. pylori* also trigger Wnt/β-catenin pathway by recruiting macrophages. Moreover, Wnt/β-catenin pathway is found involved in *H. pylori*-induced gastric cancer stem cell generation. Recently, by using gastroids, researchers have further revealed that *H. pylori* induce gastric epithelial cell proliferation through β-catenin. These findings indicate that Wnt/β-catenin is an oncogenic pathway activated by *H. pylori*. Therefore, this pathway is a potential therapy target for *H. pylori*-related gastric cancer.

## INTRODUCTION

**Figure 1:**
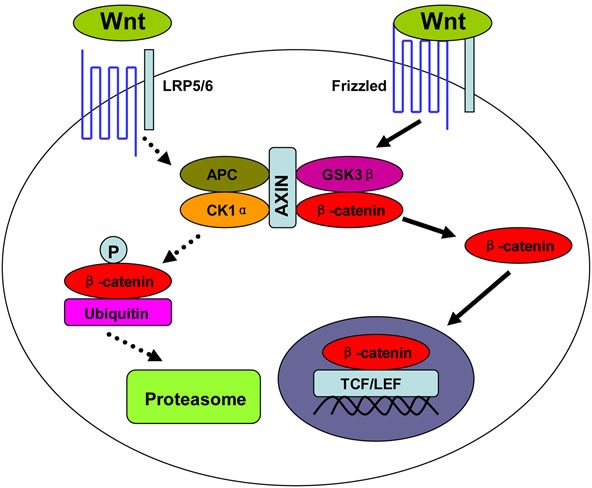
Wnt/β-catenin signal pathway Upon the binding of Wnt proteins to their receptors, β-catenin dissociates from its degrading complex, which consists of scaffold protein AXIN, casein kinase 1α (CK1α), tumor suppressor adenomatous polyposis coli (APC), and glycogen synthase kinase 3β (GSK3β). The accumulated β-catenin in cytoplasm then translocates into nucleus. P: Phosphorylation.

Wnt/β-catenin pathway, also called canonical Wnt pathway, is crucial to embryo development and adult tissue homeostasis [1, 2]. Aberrant activation of this pathway can cause uncontrolled cell growth and cell malignant transformation [1, 2]. This oncogenic pathway is initiated by some secreted glycoproteins, such as Wnt1 and Wnt3a. The binding of these Wnt proteins to their membrane receptor Frizzled and co-receptor lipoprotein receptor-related protein 5/6 (LRP5/6) leads to the dissociation of β-catenin from its degrading complex. Thereafter, β-catenin escapes from phosphorylation by glycogen synthase kinase 3β (GSK3β) and subsequent degradation by ubiquitin-proteasome system (UPS). The accumulated β-catenin in the cytoplasm translocates into the nucleus, and combines with transcription factor T cell factor/lymphocyte enhancer factor (TCF/LEF) (Figure 1).

**Figure 2:**
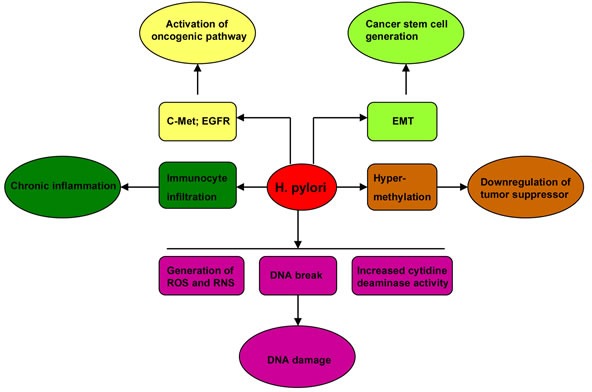
The mechanisms underlying gastric carcinogenesis induced by *H. pylori* The mechanisms include chronic inflammation in gastric mucosa, genetic and epigenetic alterations of tumor suppressor genes, activation of oncogenic signals, and generation of gastric cancer stem cells (CSC).

*Helicobacter pylori* (*H. pylori*) infection is a strong risk factor for gastric cancer. The underlying mechanisms include chronic inflammation in gastric mucosa, genetic and epigenetic alterations of tumor suppressor genes, activation of oncogenic signals, and generation of gastric cancer stem cells (CSC) (Figure 2). Chronic inflammation has been recognized as a hallmark of cancer in the recent decade [3, 4]. Aberrant activation of immune cells and overproduction of inflammatory cytokines promote gastric cancer development [5-8]. Infection with *H. pylori* can induce gastric pre-malignancies by recruiting bone marrow-derived cells (BMDCs) [9, 10]. *H. pylori* can cause DNA double-strand breaks directly [11], and cause DNA damage indirectly by stimulating the generation of reactive oxygen species (ROS) and reactive nitrogen species (RNS) [12, 13] or by increasing the activity of cytidine deaminase [14]. Hypermethylation as well as subsequent downregulation of tumor suppressor genes is an important epigenetic mechanism in *H. pylori*-related gastric carcinogenesis [15]. *H. pylori* induce gastric epithelial cell epithelial-mesenchymal transition (EMT), and generate potential cancer stem cells [16, 17]. *H. pylori* also stimulate some oncogenic pathways. Activation of epidermal growth factor receptor (EGFR) can resist *H. pylori*-induced gastric epithelial cell apoptosis [18, 19]. Moreover, increasing evidence has indicated that Wnt/β-catenin pathway is implicated in *H. pylori*-induced gastric carcinogenesis.

## NUCLEAR B-CATENIN ACCUMULATION IN GASTRIC CANCERS

Investigations on gastric cancer specimens showed that about 20%∼30% gastric cancers presented nuclear β-catenin accumulation [20, 21]. Some mutations were identified in *β-catenin* exon 3 that encodes serine-threonine phosphorylation sites for the GSK3β [20, 21]. These mutations protect β-catenin from phosphorylation by GSK3β and degradation by UPS. However, most of gastric cancer specimens with nuclear β-catenin accumulation did not harbor *β-catenin* mutation [20, 21]. In colon cancer, APC expression was frequently downregulated, leading to the disassembly of β-catenin degradation complex [22]. Unlike colon cancer, neither *APC* mutation [23, 24] nor *APC* methylation [25] seemed to be involved in gastric cancer. These findings suggest that other factors are involved in Wnt/β-catenin activation in gastric cancers.

Several studies from a same group demonstrated the attribution of *H. pylori* to nuclear β-catenin accumulation. Nuclear β-catenin mainly localized in epithelial cells within the proliferative zone in antral glands, and appeared more frequently in *H. pylori* cytotoxin-associated gene A (CagA)-positive specimens, compared with either CagA-negative or uninfected patients [26]. CagA-positive *H. pylori* could induce nuclear β-catenin accumulation *in vivo* and *in vitro* [26-28]. Recently, the group further revealed that *H. pylori* promoted gastric epithelial cell proliferation through β-catenin by using gastroids, three-dimensional organ-like structures [29].

## ACTIVATION OF ONCOGENIC C-MET AND EGFR BY H. PYLORI

Aberrant activation of c-Met receptor occurred commonly in gastric cancers [30]. Infection with CagA-positive *H. pylori* induced phosphorylation of c-Met and gastric epithelial cell proliferation [31, 32]. Upon translocating into the cytoplasm, CagA combined with c-Met and CD44 to form a functional complex [31, 32]. CD44 deficiency or inhibition blocked *H. pylori*-induced gastric epithelial cell proliferation and atrophic gastritis [32]. Activation of c-Met triggered phosphatidylinositol 3-kinase (PI3K)/Akt signaling and caused β-catenin accumulation [33]. C-Met-PI3K-β-catenin pathway is also involved in colorectal cancer. Activation of this pathway promoted cell invasion and proliferation, and protected cells from apoptosis [34]. On the contrary, inactivation of c-Met augmented GSK3β activity and β-catenin degradation [35]. Interestingly, β-catenin accumulation could also upregulate c-Met expression [35, 36], indicating a positive feedback between c-Met and β-catenin in carcinogenesis.

**Figure 3:**
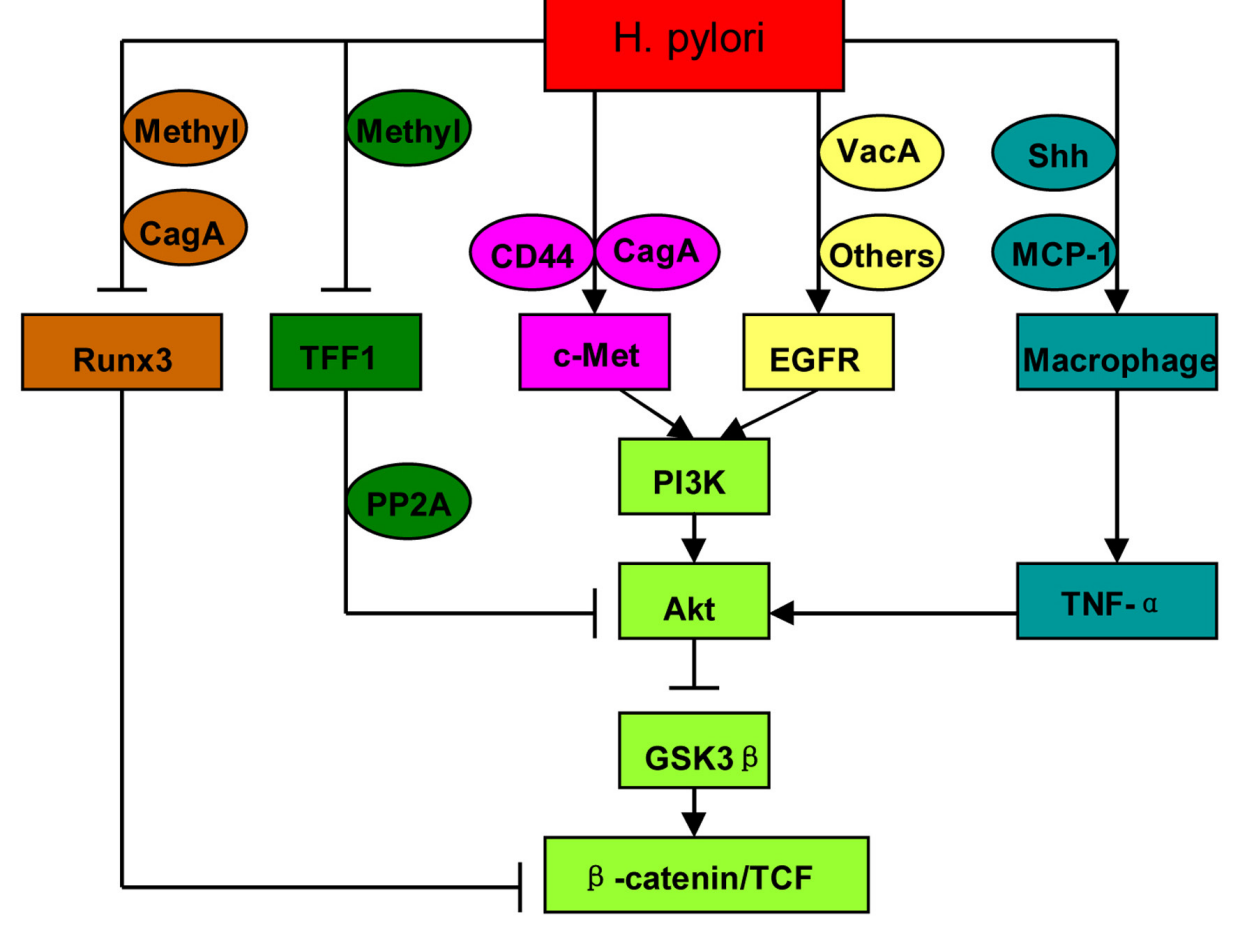
Intracellular signalings mediating the activation of Wnt/β-catenin by *H. pylori* Methyl: methylation.

EGFR signals another oncogenic pathway in *H. pylori*-related gastric cancer [18, 19]. Unlike c-Met, activation of EGFR involved vacuolating cytotoxin A (VacA) [37], CagE [38], CagL [39], *H. pylori* secretory protein HP0175 [40], and outer inflammatory protein A (OipA) [41], whereas not CagA. Indeed, CagA inactivated EGFR by activating SH2 domain-containing protein tyrosine phosphatase (SHP-2) [42]. *H. pylori* induced EGFR phosphorylation, and then activated PI3K/Akt pathway [19]. Activation of EGFR-PI3K/Akt signaling resulted in GSK3β suppression and β-catenin accumulation via VacA or OipA [41, 43, 44]. These observations indicate that intracellular pathways initiated by EGFR and c-Met converge at PI3K/Akt-GSK3β-β-catenin under *H. pylori* infection (Figure 3).

## DOWNREGULATION OF TUMOR-SUPPRESSOR RUNX3 AND TFF1 BY H. PYLORI

Runx3 is an important tumor suppressor for gastric cancer. *Runx3* expression was frequently downregulated in gastric cancer cells because of promoter hypermethylation. The clinicopathological analysis on gastric cancers and premalignant lesions showed that *Runx3* hypermethylation was correlated with *H. pylori* infection [45, 46]. In addition to gene hypermethylation, other mechanisms are also involved in *H. pylori*-induced Runx3 downregulation. CagA could directly associate with Runx3 through a specific recognition of the PY motif of Runx3 by a WW domain of CagA, and result in the ubiquitination and degradation of Runx3 [47]. CagA could also reduce *Runx3* mRNA expression by inhibiting *Runx3* promoter activity [48]. Runx3 suppressed Wnt/β-catenin pathway by forming a ternary complex with β-catenin/TCF4 [49]. Therefore, Runx3 loss upregulated the expression of Wnt/β-catenin target genes, and induced gastric carcinogenesis [50] (Figure 3).

Trefoil factor 1 (TFF1) was expressed in normal gastric mucosa [51], but frequently downregulated in gastric cancers because of gene mutation [52] and promoter hypermethylation [53]. Recombinant TFF1 protein inhibited gastric epithelial cell proliferation, whereas mutant TFF1 protein lost this effect [54]. Moreover, animals with TFF1 inactivation developed gastric pre-malignant lesions and gastric cancer [55]. These studies indicate that TFF1 is a crucial tumor suppressor for gastric cancer. It is unclear whether *H. pylori* can induce *TFF1* gene mutation, but there is evidence suggesting that *H. pylori* are responsible for *TFF1* gene hypermethylation. TFF1 was significantly downregulated and frequently methylated in *H. pylori*-positive mucosa, compared with *H. pylori*-negative mucosa [53, 56]. In N-methyl-N-nitrosourea (MNU)-induced gastric cancers, *TFF1* methylation was increased after *H. pylori* infection [53]. TFF1 inhibited Akt and GSK3β phosphorylation through protein phosphatase 2A (PP2A), and then reduced β-catenin nuclear translocation and TCF transcription activity [57]. On the contrary, TFF1 loss promoted *H. pylori*-induced oncogenic activation of β-catenin [58] (Figure 3).

## MACROPHAGES CONNECTING INFLAMMATION WITH WNT/B-CATENIN ACTIVATION

It has been well accepted that tumor-associated macrophages (TAMs) promote cancer development. *H. pylori* infection recruited macrophages via monocyte chemoattractant protein-1 (MCP-1) [7, 59] or Sonic Hedgehog (Shh) [60] in gastric mucosa. These macrophages produced pro-inflammatory cytokines, such as TNF-α and IL-1β. TNF-α could activate Wnt/β-catenin via Akt-GSK3β signaling in gastric cancer [6, 7] (Figure 3). Macrophage-derived IL-1β inhibited GSK3β activity and β-catenin degradation, and enhanced TCF transcription activity in colon cancers [61]. The suppression of GSK3β by IL-1β depended on NF-κB and Akt activation [62]. Macrophages are also involved in Wnt/β-catenin activation in cholangiocarcinoma [63]. These observations demonstrate macrophages as important linkers between chronic inflammation and Wnt/β-catenin activation.

## MICRORNAS: POTENTIAL LINKERS BETWEEN H. PYLORI INFECTION AND WNT/B-CATENIN ACTIVATION

MicroRNAs (miRs) are small noncoding RNAs that can up- or downregulate the expression of oncogenes and tumor suppressors. Some miRs, such as miR-101, mir-124a, miR-203, miR-210 and miR-320, were downregulated by *H. pylori*. MiR-101 and miR-320 were inhibited by *H. pylori* through CagA [64, 65]. Hypermethylation was responsible for miR-124a, miR-203 and miR-210 downregulation [66-68]. The reduction in expression of these miRs activated Wnt/β-catenin pathway in different cells or tissues [69-73], indicating that these miRs functioned as tumor suppressors. On the contrary, miR-21, miR-155, and miR-222 were upregulated by *H. pylori* [74-76]. These miRs stimulated Wnt/β-catenin pathway, and functioned as oncogenes or tumor-promoters [77-79]. The implication of these miRs in Wnt/β-catenin pathway in gastric cancer remains unknown. Actually, these miRs are candidates linking *H. pylori* infection with Wnt/β-catenin activation in gastric cancer (Table 1).

**Table 1:** Potential microRNAs linking H. *pylori* infection with Wnt/β-catenin activation

miR	Regulation by *H. pylori*	Reference	Effect on Wnt/β-catenin	Cell/Tissue	Reference
21	up	74	activation	colorectal cancer	77
101	down	64	inhibition	colorectal cancer	69
124a	down	66	inhibition	glioma	71
155	up	75	activation	hepatocellular carcinoma	78
203	down	67	inhibition	breast cancer	72
210	down	68	inhibition	adipose	73
222	up	76	activation	breast cancer	79
320	down	65	inhibition	prostate cancer	70

## THE EFFECTS OF H. PYLORI ON UPSTREAM MOLECULES IN WNT/B-CATENIN PATHWAY

Some evidence indicates that *H. pylori* may activate Wnt/β-catenin pathway by affecting Wnt ligands, receptors or antagonists. *H. pylori* and TNF-α could induce Wnt10a and Wnt10b expression in gastric cancer cells [80, 81]. *H. pylori* infection could also activate Wnt co-receptor LRP6, and result in nuclear β-catenin accumulation [82]. Secreted Frizzled-related proteins (SFRPs) can combine with Wnt ligands or receptors to interfere Wnt signaling. These Wnt antagonists were frequently downregulated in gastric cancers due to gene promoter hypermethylation [83]. Actually, *SFRP4* and *SFRP5* methylation was found to be positively correlated with *H. pylori* infection [84]. In addition, Wnt3 [85], Wnt7a [86], Wnt7b [87] and Wnt receptor Frizzled [88], were also expressed in gastric cancer cells. The effects of *H. pylori* on these molecules are still unclear.

## THE ROLE OF WNT/B-CATENIN PATHWAY IN H. PYLORI-INDUCED GASTRIC STEM CELL GENERATION AND EXPANSION

Gastric stem cells are implicated in gastric cancer initiation and progression. Via CagA, *H. pylori* colonized stomach gland epithelium, and promoted stem cell-related gene expression and Lgr5(+) stem cell proliferation [89]. *H. pylori* also induced gastric epithelial cell EMT to generate gastric cancer stem cells, and this process was also via CagA [16]. The molecular mechanisms underlying *H. pylori*-induced EMT and stem cell generation remain largely unknown. Wnt/β-catenin pathway was important for gastrointestinal progenitor cell proliferation and differentiation [90, 91]. Activation of this pathway could induce EMT in gastric cancer [92, 93]. Recently, it was revealed that CagA induced EMT by inhibiting GSK-3 activity [94]. Moreover, Wnt/β-catenin target CD44 was observed to be needed in *H. pylori*-induced gastric stem cell proliferation [95]. These findings indicate that *H. pylori* induce gastric stem cell generation and proliferation at least partly via Wnt/β-catenin pathway.

## CONCLUSION

Increasing evidence demonstrates Wnt/β-catenin as a crucial pathway stimulated by *H. pylori* in gastric carcinogenesis. *H. pylori* can upregulate Wnt/β-catenin activator c-Met and EGFR, and downregulate Wnt/β-catenin suppressor TFF1 and RUNX3. *H. pylori* can also activate Wnt/β-catenin pathway by recruiting tumor-associated macrophages. Importantly, via Wnt/β-catenin pathway, *H. pylori* induced gastric stem cell generation and expansion, promoting gastric cancer initiation and progression.

However, there are still some questions need to be answered. Which signal molecule plays a dominant role in Wnt/β-catenin activation under *H. pylori* infection, c-Met, EGFR, TFF1, Runx3, or else? Can dysregulations of these molecules synergize in gastric cancer development? Which virulent factor of *H. pylori* plays a dominant role in Wnt/β-catenin activation, CagA, VacA or else? Given the complexities of *H. pylori* strains and host factors, more work should be done to find the answers. In addition, the effects of *H. pylori* on Wnt ligands, receptors and antagonists, and the roles of miRs in Wnt/β-catenin activation in gastric cancer, need to be further investigated.

Recently, a series of therapies antagonizing Wnt/β-catenin pathway have entered clinical trials. As Wnt/β-catenin pathway is essential for tissue homeostasis, it remains elusive about their clinical efficacy and safety [96]. *H. pylori* eradication can reduce the risk of gastric cancer, but it can not completely prevent *H. pylori*-related gastric carcinogenesis. One of the reasons is that the activation of oncogenic pathway, such as Wnt/β-catenin, has happened before *H. pylori* eradication.
